# Association mapping of resistance to emerging stem rust pathogen races in spring wheat using genotyping‐by‐sequencing

**DOI:** 10.1002/tpg2.20050

**Published:** 2020-09-23

**Authors:** Erena A. Edae, Matthew N. Rouse

**Affiliations:** ^1^ Department of Plant Pathology University of Minnesota St. Paul MN 55018 USA; ^2^ USDA‐ARS Cereal Disease Laboratory 1551 Lindig Street St. Paul MN 55018 USA

## Abstract

The identification and characterization of resistance genes should outpace the rapid emergence of new *P. graminis* f. sp. *tritici* races, such as TTRTF and TTKTT, to mitigate stem rust damage to wheat. The objective of the current study was to identify and characterize *P. graminis* f. sp. *tritici* race resistance association signals. A total of 250 North American spring wheat lines were evaluated at the seedling stage with a total of seven isolates including TKKTP, TKTTF, TKTTF, TRTTF, TTRTF, TTKSK, and TTKTT. The lines were genotyped by a GBS platform and 9,042 SNPs were used for identification of chromosome regions associated with resistance against the seven isolates. Strong association signals were detected on chromosomes 6BL (*Sr11* gene region) and 4AL, likely *Sr7a*, for resistance against both TKKTP and TKTTF. Similarly, association signals were also detected on chromosomes 4AL (race TTRTF resistance) and 4BS (race TTKSK and TTKTT resistance). Association analysis based on mean phenotypic differences between closely related isolates identified QTL that were not elucidated by direct association mapping of the responses, individually. Overall, with the exception of race TRTTF, each race shared at least one association signal with another race. However, the number of race‐specific association signals are larger than that of association signals common among races suggesting the need for identifying and characterizing QTL/genes for newly emerging stem rust pathogen races. There was also high concordance between PCA‐based GWAS association signals and association signals from that of both single and multi‐locus mixed models.

**Abbreviations**
BLUEs: Best linear unbiased Estimators
GBS: Genotype‐by‐sequencing
LD: Linkage Disequilibrium
MLMM: multi‐locus mixed model
MTA: Marker‐trait association
PCA: Principal component analysis
*Pgt*
: *Puccinia graminis* f. sp. *tritici*
SLMM: single‐locus mixed linear model
SNP: single nucleotide polymorphisms
TCAP: *Triticeae* Coordinated Agricultural Project.

## 1 INTRODUCTION

Wheat stem rust, caused by the fungus *Puccinia graminis* f. sp. *tritici* (*Pgt*), has been a major threat to the production of wheat at the global scale. Although stem rust damage to wheat production can be controlled by chemicals, this approach can be difficult to implement in countries with limited access to agricultural chemicals. Alternately, wheat stem rust can be controlled effectively through developing stem rust resistant varieties.

In the past 50 years several stem rust resistance (*Sr*) genes have been discovered and effectively utilized to control stem rust. For instance, the deployment of gene *Sr31* into modern wheat cultivars starting in 1960 successfully and sustainably controlled the disease until the emergence of a new *Pgt* race named Ug99 (TTKSK; Pretorius, Singh, Wagoire, & Payne, 2000) in Uganda. Stem rust pathogen races in the Ug99 race group threaten global wheat production because they are virulent on the majority of deployed *Sr* genes that had been effectively utilized for many years (Singh et al., 2006). The Ug99 race group threatens more than 80% of worldwide wheat production (Singh et al., 2011). Moreover, subsequent monitoring of *Pgt* races in East Africa indicated that members of the Ug99 race group are evolving rapidly, and there are now 13 reported variants, most of which are virulent to genes that were resistant to the original race TTKSK (Jin et al., 2008; Jin et al., 2009; Newcomb et al., 2016). The Ug99 race group has also already spread to countries outside Africa such as Yemen, and Iran, where it is now poised to threaten wheat production in South Asia. Because of the constant evolution and mutation in *Pgt*, new virulent races that are not in the Ug99 race group have been observed in recent years. Recently, Li et al. (2019) reported that somatic hybridization, and multiple nuclear exchange events between *Pgt* strains have contributed to the genetic diversity in global *Pgt* populations.

Race TKTTF that caused a severe epidemic in the Southeastern part of Ethiopia in 2013–2014 is not a member of the Ug99 race group (Olivera et al., 2015). This race was recently reported in several countries including in European countries such as Sweden, Denmark, and Germany (Olivera et al., 2017; Rahmatov et al., 2016). Genes that provide resistance to race TKTTF include *Sr7a*, *Sr11*, and *Sr31*. Other races were reported from Germany including a race TKKTP with virulence to several Ug99‐effective genes including *SrTmp, Sr24*, and *Sr1RS^amigo^* (Olivera et al., 2017). *Pgt* race TRTTF was detected in Yemen in 2006 and subsequently in Ethiopia. It is virulent to wheat stem rust resistance genes such as *Sr1RS^amigo^*, *Sr36*, and *SrTmp* (Olivera et al., 2012). However, genes such as *Sr8a, Sr22, Sr24, Sr26, Sr27, Sr31, Sr32, Sr33, Sr35, Sr39, Sr40, Sr46, Sr47*, and *Sr50* confer resistance to race TRTTF (Olivera et al., 2012). A race TTRTF (initially reported as TTTTF) caused an epidemic of stem rust on wheat in Sicily in 2016 (Bhattacharya et al., 2017). Race TTRTF possesses virulence to *Sr13b*, a gene common in durum wheat (Zhang et al., 2017), and several other resistance genes (Bhattacharya et al., 2017). The virulence of this race on conventional wheat germplasm has not been reported.

Since the discovery of Ug99, the search for resistance genes has examined both domesticated wheat and its wild relatives. There are 32 numerically designated stem rust resistance genes that are effective to the Ug99 race group (Rouse, Talbert, Singh, & Sherman, 2014b; Singh et al., 2015): *Sr2*, *Sr12*, *Sr13*, *Sr14*, *Sr15*, *Sr22*, *Sr25*, *Sr26*, *Sr27*, *Sr28*, *Sr29*, *Sr32*, *Sr33*, *Sr35*, *Sr37*, *Sr39*, *Sr40*, *Sr42*, *Sr43*, *Sr44*, *Sr45*, *Sr46*, *Sr47*, *Sr48*, *Sr50*, *Sr51*, *Sr52*, *Sr53*, *Sr55*, *Sr56*, *Sr57*, and *Sr58*. Of these genes, relatively few are available in conventional North American spring wheat germplasm including *Sr2, Sr13, Sr25, Sr26, Sr55*, and *Sr57* (Bajgain et al., 2015a; Edae, Pumphrey, & Rouse, 2018). Many QTL have also been reported using bi‐parental populations (Haile, Nachit, Hammer, Badebo, & Roder, 2012; Hiebert, Fetch, & Zegeye, 2010; Kumsa et al., 2015; Njau, Bhavani, Huerta‐Espino, Keller, & Singh, 2013; Zurn et al., 2014; Bajgain et al., 2015b), and association mapping (Bajgain et al., 2015a; Edae et al., 2018; Letta et al., 2013; Yu et al., 2011; Zhang, Bowden, Jin, Carver, & Bai, 2014). This effort of identifying new resistance genes needs to continue in order to mitigate the effects of the constantly evolving pathogen. Therefore, in the current work, we identified chromosome regions associated with emerging races of *Pgt* using GBS SNP markers.

**Core Ideas**

- Response of conventional North American spring wheat to emerging races of the wheat stem rust pathogen was reported.
- Chromosome regions associated with resistance against emerging wheat stem rust pathogen races were identified.
- Phenotypic difference mapping resolved resistance loci effective to new variant races and specific to particular temperatures.
- PCA‐GWAS identified loci that confer resistance against stem rust pathogen races.

## 2 MATERIALS AND METHODS

### 2.1 Plant materials and phenotyping

The spring wheat association mapping panel used in this study was assembled by the *Triticeae* Coordinated Agricultural Project (TCAP) and consisted of 250 accessions from spring wheat breeding programs in the United States, Canada, and the International Maize and Wheat Improvement Center (CIMMYT) (Edae et al., 2018). We screened the panel with five non‐Ug99 *Pgt* races including TKKTP (isolate 13GER16‐1; Olivera et al., 2015), TKTTF (13ETH18‐1; Olivera et al., 2015), TKTTF (13GER10‐5; Olivera et al., 2015), TRTTF (06YEM34‐1; Olivera et al., 2012), TTRTF (14GEO189‐1; Olivera et al., 2019), and Ug99 variants TTKSK (04KEN156/04; Jin & Singh, 2006), and TTKTT (14KEN58‐1; Newcomb et al., 2016)). In this manuscript we distinguish the two isolates of race TKTTF by an abbreviation for each: TKTTF‐ETH (13ETH18‐1), and TKTTF‐GER (13GER10‐5). The races were evaluated in a greenhouse for seedling responses at the USDA‐ARS Cereal Disease Laboratory, St. Paul, MN according to methods described by Hundie et al. (2019). Race TTKSK was screened both in the greenhouse under a normal temperature (19–22 °C), and a cold temperature regime (15 °C to 18 °C, night/day, with a 16 h photoperiod) in a growth chamber. Infection types (ITs) were recorded according to the 0–4 scale (Stakman, Stewart, & Loegering, 1962), where “0”, “;”, “1”, and “2” indicate resistance and “3” and “4” indicate susceptibility. Relatively smaller and larger pustule sizes for each IT class were noted with “−” and “+” symbols, respectively. When two or more ITs were present on the same leaf, all ITs were recorded; with the most frequent IT listed first. When a line was heterogeneous for IT, a “/” symbol was used to separate ITs recorded for different plants. The 0–4 scale data were converted to a 0–9 linear scale for subsequent analysis (Gao, Turner, Chao, Kolmer, & Anderson, 2016). For the linear scale, values of 6 or lower indicated resistance, corresponding to 2+ or lower on the 0–4 scale. Each race was evaluated under two replications. The races were assessed on the germplasm between the year 2013 and 2019. The seedling data of each race were subjected to analysis of variance using SAS v. 9.3 software (SAS Institute, 2011). Best linear unbiased Estimators (BLUEs) were used for GWAS analysis.

### 2.2 SNP genotyping

In order to obtain SNP markers without any ascertainment bias, a total of 248 accessions in the spring wheat association panel were genotyped with the wheat GBS platform (Poland et al., 2012). The GBS libraries were constructed in 96‐plex following co‐digestion with two restriction enzymes *Pst*I (CTGCAG) and *Msp*I (CCGG) followed by barcoded adaptors ligation of individual samples. The samples were pooled per plate and PCR‐amplified (Poland et al., 2012). Each library was sequenced on the Illumina HiSeq 2000 platform using single end sequencing from *Pst*1 sites. The SNPs were called using hexaploid wheat reference v1.0 (International Wheat Genome Sequencing Consortium, 2018). All SNPs with missing values above 25%, heterozygosity greater than 5% and minor allele frequency (MAF) less than 5% were removed. The final filtered data set consisted of 9,042 SNPs for 248 accessions. The remaining missing data points were imputed with the LD‐KNNI imputation algorithm (Money et al., 2015) in TASSEL 5 (Bradbury et al., 2007). For the purpose of identifying genetic positions of trait‐associated markers, SNP data were integrated into POPSEQ data of Chinese Spring.

### 2.3 Population structure and kinship analyses

The pattern of population structure was previously reported for this population (Bajgain et al., 2015a; Edae et al., 2018) using Illumina Infinium iSelect SNP chip for wheat (Illumina Inc., Hayward, CA, USA; Wang et al., 2014). Population structure was also assessed with GBS‐based SNPs in the current work using principal component analysis using prcomp command in R (R Core Team, 2016).

### 2.4 Linkage disequilibrium (LD) analysis

Pairwise LD was calculated among 9,042 SNP markers as squared allele frequency correlation (r^2^) in Synbreed R package (http://synbreed.r-forge.r-project.org/). LD decay curves were also computed for chromosome arms 6BL and 7BL on which strong association signals were detected for resistance against three *Pgt* races (TTKSK, TKKTP and TKTTF).

### 2.5 Genome‐wide association analysis

To identify chromosome regions associated with stem rust resistance, a single‐locus mixed linear model (SLMM) (Yu et al., 2006) that was implemented in GAPIT R package (Lipka et al., 2012) was used with a compressed mixed linear model that involved clustering of individuals into groups using clustering algorithms (CMLM) (Li et al., 2014; Tang et al., 2016). The multi‐locus mixed model (MLMM) developed by Segura et al. (2012) was used on the same data set to identify the most significant marker‐trait associations. The extended Bayesian information criterion (EBIC) was used for model selection criteria in the MLMM. Both SLMM and MLMM analyses were included as a form of validation of the results from both methods as has been effectively used in other studies of wheat stem rust resistance association mapping (Edae et al., 2018; Mihalyov, Nichols, Bulli, Rouse, & Pumphrey, 2017). In addition to validation of results from SLMM, the MLMM proved useful for detecting QTL that would not have been detected without taking into account the most significant QTL (Mihalyov et al., 2017). Population structure using the first 10 principal components and genetic relatedness among individuals (K) were fitted as covariates in both mixed models except for races TKTTF‐GER and TTKSK evaluated under cold temperature for which only population structure was included in the models. This decision was based on how well the model used in GWAS accounts for population structure and familial relatedness using quantile‐quantile (Q‐Q) plot of p‐values. A marker‐wise p‐value of .0001 was used to declare significant QTL in the SLMM and MLMM. The GWAS analysis was also conducted for mean difference of infection type for race TTKSK under normal temperature and cold temperature, mean difference in infection type between race TTKSK and TTKTT, and mean difference in infection type for TKTTF from Ethiopia (TKTTF‐ETH) and TKTTF from Germany (TKTTF‐GER). Mean difference in infection type was calculated by determining the difference in BLUE values of the pairs of races for each line.

Principal component analysis was applied to identify loci that are effective against all *Pgt* races. Phenotypic responses of the test materials for all *Pgt* races were subjected to Principal component analysis and the scores of each PC were used as a trait in GWAS. The loadings from PCA which represent the correlation between variables and PCs were extracted to understand the relationship between Marker‐trait associations (MTAs) detected with each PC and response to the individual *Pgt* races.

## 3 RESULTS

### 3.1 Stem rust response

**FIGURE 1:**
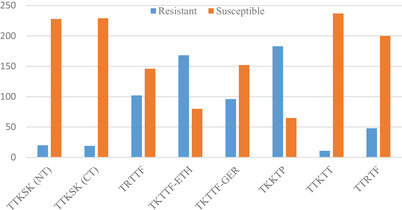
Proportion of resistant and susceptible spring wheat lines to *Puccinia graminis* f. sp. *tritici* races. NT, normal temperature; CT, cold temperature

**FIGURE 2:**
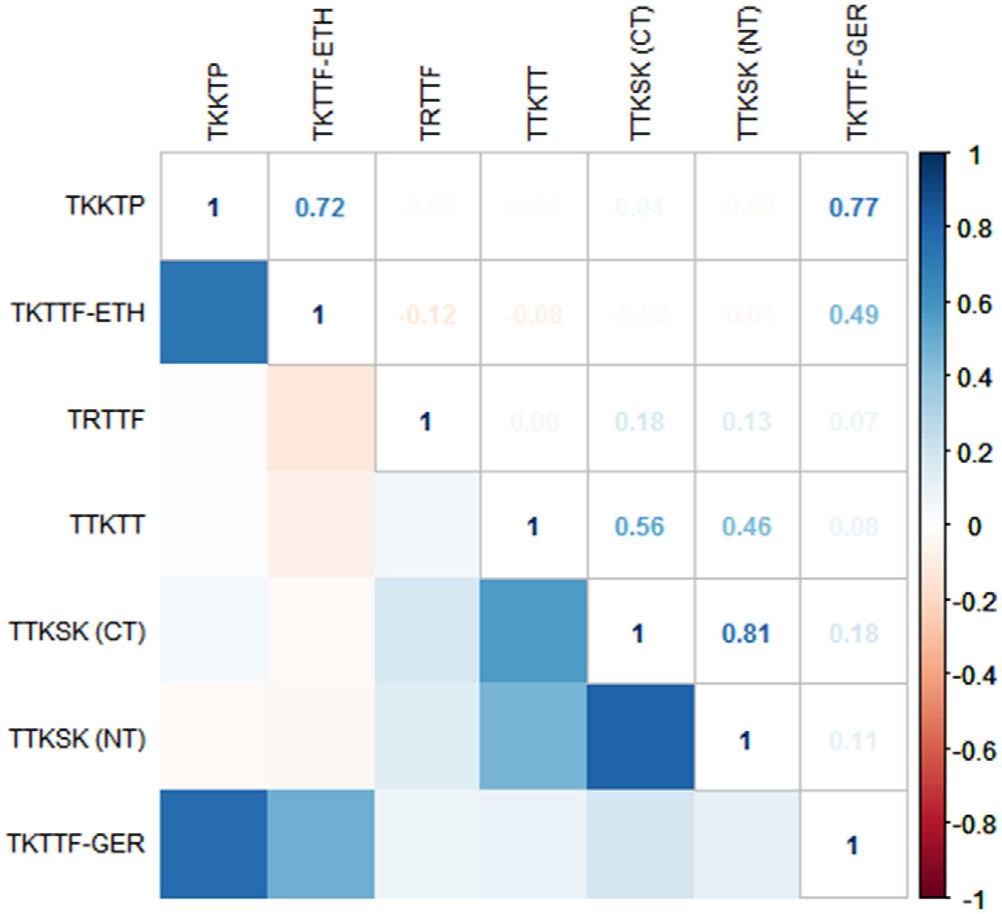
Phenotypic correlation coefficients among *Puccinia graminis* f. sp. *tritici* races. Values between −0.1 and 0.1 were not significant and thus may not be visible

The percentage of resistant lines varied from 4.84% to 8.07% for Ug99 race group variants TTKTT and TTKSK. The percentage of resistant lines for the remaining *Pgt* races varied from 19.76% (race TTRTF) to 74.19% (TKKTP) suggesting Ug99 race variants are more virulent than non‐Ug99 races on conventional North American germplasm (Figure 1). The largest phenotypic correlation was between TTKSK data under normal and cold temperature conditions (r = 0.81) followed by the correlation coefficient of race TKKTP with races TKTTF‐GER and TKTTF‐ETH (Figure 2).

### 3.2 Genome‐wide marker distribution, population structure and LD analysis

The 9,042 GBS markers were not evenly distributed across the 21 chromosomes of hexaploid wheat. Chromosomes 2B and 7A included the largest number of markers while chromosome 4D had the least number (Supplemental Figure S1). Generally, the D genome chromosomes comprised less markers compared to the A and B genomes. Two groups within the population were previously reported based on 90K SNP data for this mapping panel (Edae et al., 2018). Similarly with GBS markers, there were also two major groups using principal component analysis (PCA; Supplemental Figure S2) representing germplasm from either the northern Great Plains or from western North America including CIMMYT lines. The LD curve crossed the r^2 ^= 0.2 line at a physical distance of 3.8 Mbp for chromosome arm 6BL (Supplemental Figure S3) while it crossed the r^2 ^= 0.2 line at a physical distance of 4.5 Mbp for 7BL (Supplemental Figure S4). A majority of the pair‐wise comparisons were weak (less than 0.1) for both chromosomes 6DL and 7BL as indicated by LD heat maps in Supplemental Figures S5 and S6, respectively.

**FIGURE 3:**
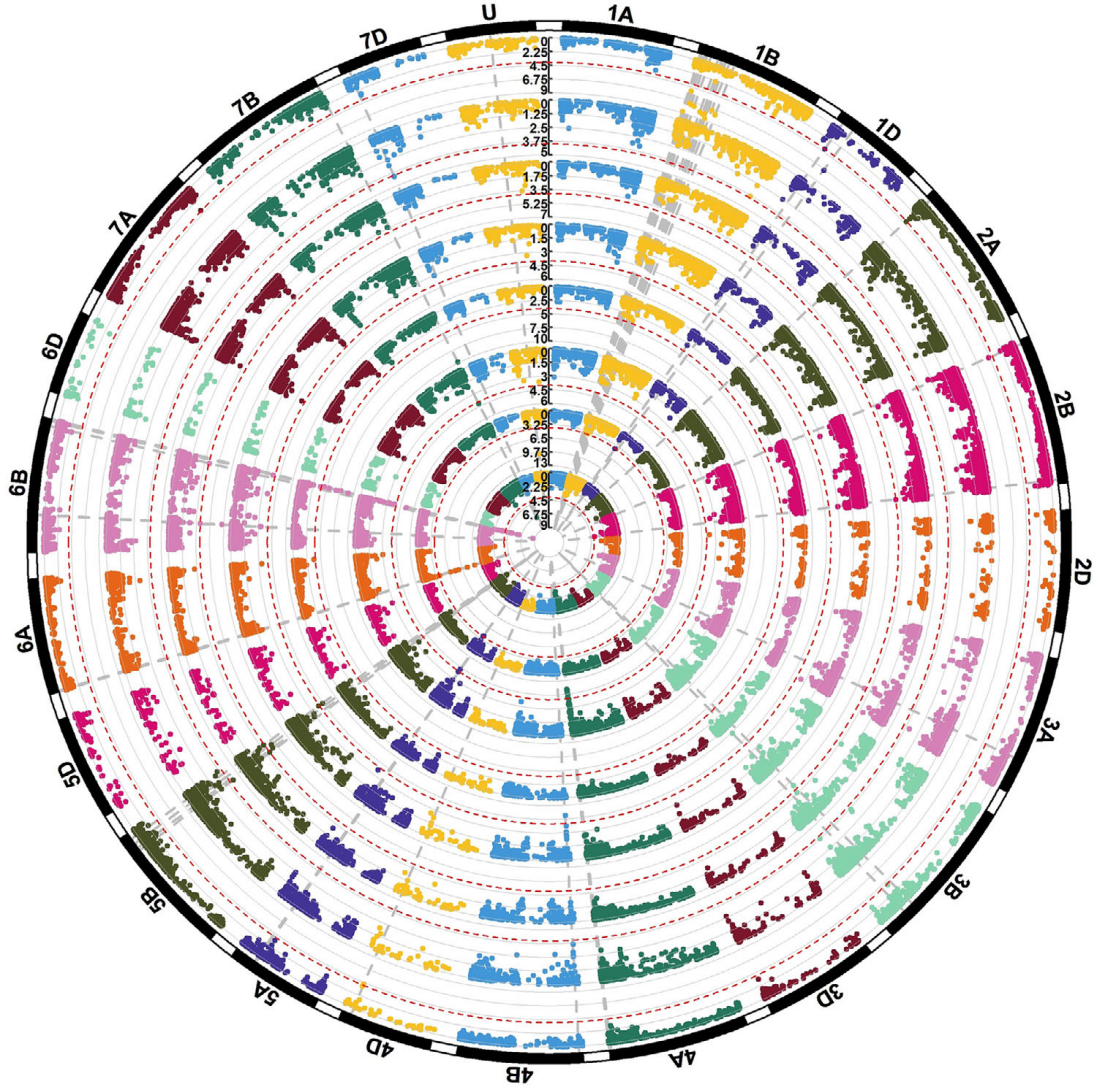
Association signals detected for *Puccinia graminis* f. sp. *tritici* races TKKTP, TRTTF, TKTTF‐ETH, TKTTF‐GER, TTKSK‐CT, TTKSK‐NT, TTKTT and TTRTF. The track number starts from inside outward with TKKTP assigned to track 1 whereas the last track (track 8) was assigned to TTRTF, respectively. The threshold p‐value (p = .0001) was indicated by dotted red line for each track

MTAs identified with the SLMM and MLMM models are given in Tables 1 and 2, respectively. Overall, 64 and 23 MTAs were detected with the SLMM and MLMM, respectively. The MTAs detected by SLMM were visualized comparatively in Figure 3. A majority of the MTAs identified corresponded to four genomic regions: chromosome 1B, likely conferred by *Sr31*; chromosome arm 4AL, likely conferred by *Sr7a*; chromosome arm 6AS, likely conferred by *Sr8a*; and chromosome arm 6BL, likely conferred by *Sr11*. These genomic regions conferred race‐specific resistance to the expected races. Each of the four regions were detected in both the SLMM and MLMM, providing some confidence in the results. In addition to these four known resistance genes, several QTL were identified to various races including seven QTL validated by both the SLMM and MLMM: chromosomes 2A and 2B, effective to TTRTF; chromosome 7B, effective to TKTTF‐GER; chromosome 4B, effective to TTKSK and TTKTT; chromosome 7D, effective to TTKTT; chromosome 3A, effective to TTKSK and TTKTT; and chromosome 5A, effective to TRTTF. The positions and p‐values of these QTL can be found in Tables 1 and 2.

**TABLE 1:** Marker‐trait associations (MTAs) detected for seven *Puccinia graminis* f. sp. *tritici* isolates corresponding to races TRTTF, TTKSK, TKTTF, TRTTF, TKKTP and TTRTF

Race^a^	SNP	Chrom	map pos (Mbp)	P‐value	MAF	FDR_p‐value	R^2^
TTRTF	S1B_PART1_15659890	1B	15.66	1.73×10^−9^	0.06	7.81×10^−6^	0.127
TTRTF	S1B_PART1_28041285	1B	28.04	7.24×10^−6^	0.14	5.456×10^−3^	0.07
TTRTF	S1B_PART1_39577562	1B	39.58	7.79×10^−8^	0.06	7.83×10^−5^	0.10
TTRTF	S1B_PART1_53250247	1B	53.25	5.70×10^−8^	0.05	6.44×10^−5^	0.10
TTRTF	S1B_PART1_74249006	1B	74.25	4.15×10^−8^	0.06	5.36×10^−5^	0.10
TTRTF	S1B_PART1_115449622	1B	115.45	1.11×10^−8^	0.06	2.00×10^−5^	0.11
TTRTF	S1B_PART1_136168911	1B	136.17	7.60×10^−9^	0.06	2.00×10^−5^	0.12
TTRTF	S1B_PART1_146796395	1B	146.80	1.11×10^−8^	0.06	2.00×10^−5^	0.11
TTRTF	S1B_PART1_158545386	1B	158.55	1.73×10^−9^	0.06	7.81×10^−6^	0.13
TTRTF	S1B_PART1_180406818	1B	180.41	9.39×10^−8^	0.05	8.49×10^−5^	0.10
TTRTF	S1B_PART1_209129922	1B	209.13	2.84×10^−8^	0.054	4.28×10^−5^	0.11
TRTTF	S1D_PART1_37956518	1D	37.96	7.19×10^−5^	0.05	0.069	0.05
TTRTF	S1D_PART1_105157874	1D	105.16	1.20×10^−7^	0.05	9.84×10^−5^	0.10
TTRTF	S2A_PART1_29263759	2A	29.26	7.53×10^−5^	0.34	0.045388	0.05
TTRTF	S2B_PART1_23965311	2B	23.97	2.14×10^−5^	0.09	0.01489	0.06
TTKSK (NT)	S2B_PART2_324160151	2B	777.38	9.81×10^−5^	0.08	0.102	0.06
TTKSK (NT)	S3A_PART2_116010925	3A	570.12	5.33×10^−5^	0.14	0.096	0.06
TTKSK (NT)	S3B_PART2_188289477	3B	636.44	2.64×10^−5^	0.47	0.06	0.07
TTKSK (CT)	S3B_PART2_251114407	3B	699.27	1.33×10^−6^	0.48	0.012	0.09
TKTTF‐ETH	S4A_PART2_280264506	4A	732.82	1.17×10^−5^	0.48	0.035	0.05
TKTTF‐ETH	S4A_PART2_280270591	4A	732.83	4.26×10^−5^	0.48	0.064	0.04
TKTTF‐ETH	S4A_PART2_286223035	4A	738.78	1.74×10^−5^	0.27	0.036	0.05
TKTTF‐ETH	S4A_PART2_290053290	4A	742.61	2.01×10^−5^	0.29	0.036	0.04
TKTTF‐ETH	S4A_PART2_290196354	4A	742.75	7.85×10^−5^	0.46	0.079	0.04
TKTTF‐ETH	S4A_PART2_290628489	4A	743.18	1.03×10^−5^	0.28	0.035	0.05
TTKSK (CT)	S4B_PART1_20568400	4B	20.57	1.28×10^−5^	0.45	0.058	0.07
TTRTF	S4D_PART2_4614180	4D	4.614	5.92×10^−5^	0.13	0.038247	0.05
TRTTF	S5A_PART2_133066686	5A	586.3	6.18×10^−5^	0.06	0.069	0.05
TRTTF	S5A_PART2_135170900	5A	588.4	6.62×10^−5^	0.06	0.069	0.05
TKTTF‐ETH	S5A_PART2_138745963	5A	591.98	4.98×10^−5^	0.22	0.064	0.04
TTKTT	S5B_PART2_150047392	5B	601.42	4.68×10^−5^	0.44	0.339	0.06
TTKSK (NT)	S5B_PART2_166247712	5B	617.62	2.49×10^−5^	0.18	0.06	0.07
TTKSK (NT)	S5B_PART2_194543855	5B	645.92	1.88×10^−5^	0.45	0.06	0.07
TTKSK (CT)	S5B_PART2_227153820	5B	678.53	3.76×10^−5^	0.48	0.113	0.06
TRTTF	S6A_PART1_3015737	6A	3.02	9.73×10^−12^	0.41	0	0.15
TRTTF	S6A_PART1_3206675	6A	3.21	1.66×10^−13^	0.49	0	0.18
TRTTF	S6A_PART1_3691352	6A	3.7	9.48×10^−9^	0.30	0	0.10
TRTTF	S6A_PART1_3705925	6A	3.71	7.62×10^−5^	0.40	0.069	0.05
TRTTF	S6A_PART1_5569284	6A	5.57	1.46×10^−7^	0.33	0	0.08
TRTTF	S6A_PART1_6842176	6A	6.82	1.46×10^−9^	0.46	0	0.11
TTKSK(NT)	S6B_PART1_201789705	6B	201.79	9.9×10^−5^	0.38	0.102	0.06
TTKSK(NT)	S6B_PART1_203291041	6B	203.29	1.81×10^−7^	0.33	0.002	0.10
TTKSK(NT)	S6B_PART2_229827112	6B	681.9	8.27×10^−5^	0.48	0.102	0.06
TKKTP	S6B_PART2_256708803	6B	708.79	4.17×10^−5^	0.33	0.047	0.05
TKTTF‐ETH	S6B_PART2_257935127	6B	710.01	7.24×10^−6^	0.23	0.035	0.05
TKTTF‐GER	S6B_PART2_257935127	6B	710.01	1.11×10^−9^	0.23	0	0.12
TKKTP	S6B_PART2_257935127	6B	710.01	2.18×10^−9^	0.23	0	0.11
TKTTF‐GER	S6B_PART2_257987924	6B	710.07	2.06×10^−7^	0.38	0	0.09
TKKTP	S6B_PART2_257987924	6B	710.07	1.79×10^−5^	0.38	0.027	0.06
TKTTF‐GER	S6B_PART2_260047154	6B	712.12	7.98×10^−8^	0.48	0	0.09
TKKTP	S6B_PART2_260047154	6B	712.13	5.01×10^−7^	0.48	0.002	0.08
TKTTF‐GER	S6B_PART2_260245119	6B	712.32	6.29×10^−5^	0.32	0.063	0.05
TKTTF‐GER	S6B_PART2_262414407	6B	714.49	4.56×10^−6^	0.43	0.006	0.07
TKKTP	S6B_PART2_262414407	6B	714.49	8.97×10^−7^	0.43	0.002	0.07
TKTTF‐GER	S6B_PART2_265342488	6B	717.42	2.95×10^−5^	0.46	0.033	0.06
TKTTF‐GER	S6B_PART2_265342731	6B	717.42	4.18×10^−6^	0.29	0.006	0.07
TKKTP	S6B_PART2_265342731	6B	717.42	7.69×10^−6^	0.29	0.014	0.06
TKTTF‐GER	S6B_PART2_267826895	6B	719.9	2.92×10^−8^	0.28	0	0.10
TKKTP	S6B_PART2_267826895	6B	719.9	3.45×10^−5^	0.28	0.045	0.05
TKTTF‐ETH	S7B_PART2_293794262	7B	745.87	6.74×10^−5^	0.36	0.076	0.04
TKTTF‐GER	S7B_PART2_293794262	7B	745.87	1.51×10^−10^	0.36	0	0.14
TKKTP	S7B_PART2_293794262	7B	745.87	1.39×10^−7^	0.36	0.001	0.09
TTKTT	S7D_PART1_45067811	7D	45.07	8.32×10^−5^	0.05	0.339	0.05
TRTTF	SUN_231626593	UN	‐	8.25×10^−11^	0.47	0	0.13

^a^ NT, Normal temperature, CT, Cold temperature; MAF, Minor allele frequency.

**TABLE 2:** Marker‐trait associations (MTAs) selected by MLMM for resistance in spring wheat to seven stem rust pathogen isolates

Race	SNP	Chrom	Position (Mbp)	p‐value
TKKTP	S6B_PART2_257935127	6B	710.01	2.41×10^−10^
TKKTP	S6B_PART2_265342731	6B	710.01	6.93×10^−5^
TKKTP	S2B_PART2_133689108	2B	586.91	4.11×10^−6^
TKKTP	S4A_PART2_280264506	4A	732.82	1.84×10^−5^
TKTTF‐GER	S7B_PART2_293794262	7B	745.87	3.32×10^−7^
TKTTF‐GER	S6B_PART2_257935127	6B	710.01	4.46×10^−6^
TKTTF‐GER	S2B_PART2_239396449	2B	692.62	0.00016
TTKSK‐CT	S7A_PART1_28119447	7A	28.12	8.88×10^−8^
TTKSK‐CT	S4B_PART1_20568400	4B	20.57	4.23×10^−7^
TTKTT	S4B_PART1_20568400	4B	20.57	4.43×10^−7^
TTKTT	S7D_PART1_42519839	7D	42.52	3.29×10^−8^
TTKTT	S3A_PART2_237279779	3A	691.38	2.65×10^−6^
TKTTF_ETH	S6B_PART2_257935127	6B	710.01	1.34×10^−9^
TKTTF_ETH	S4A_PART2_290628489	4A	743.18	6.39×10^−8^
TKTTF_ETH	S4B_PART2_210617604	4B	661.63	5.69×10^−7^
TRTTF	S6A_PART1_3206675	6A	3.21	7.95×10^−10^
TRTTF	S6A_PART1_2408343	6A	2.41	2.39×10^−11^
TRTTF	S5A_PART2_135170900	5A	588.4	7.22×10^−7^
TRTTF	S6A_PART1_6842176	6A	6.842	1.14×10^−5^
TTRTF	S1B_PART1_15659890	1B	15.66	1.12×10^−11^
TTRTF	S2B_PART1_23965311	2B	23.97	8.15×10^−6^
TTRTF	S2A_PART1_30330014	2A	30.33	1.44×10^−5^
TTRTF	S4A_PART2_268332600	4A	720.89	7.98×10^−5^

#### 3.2.1 Mean infection type difference‐based MTAs

**FIGURE 4:**
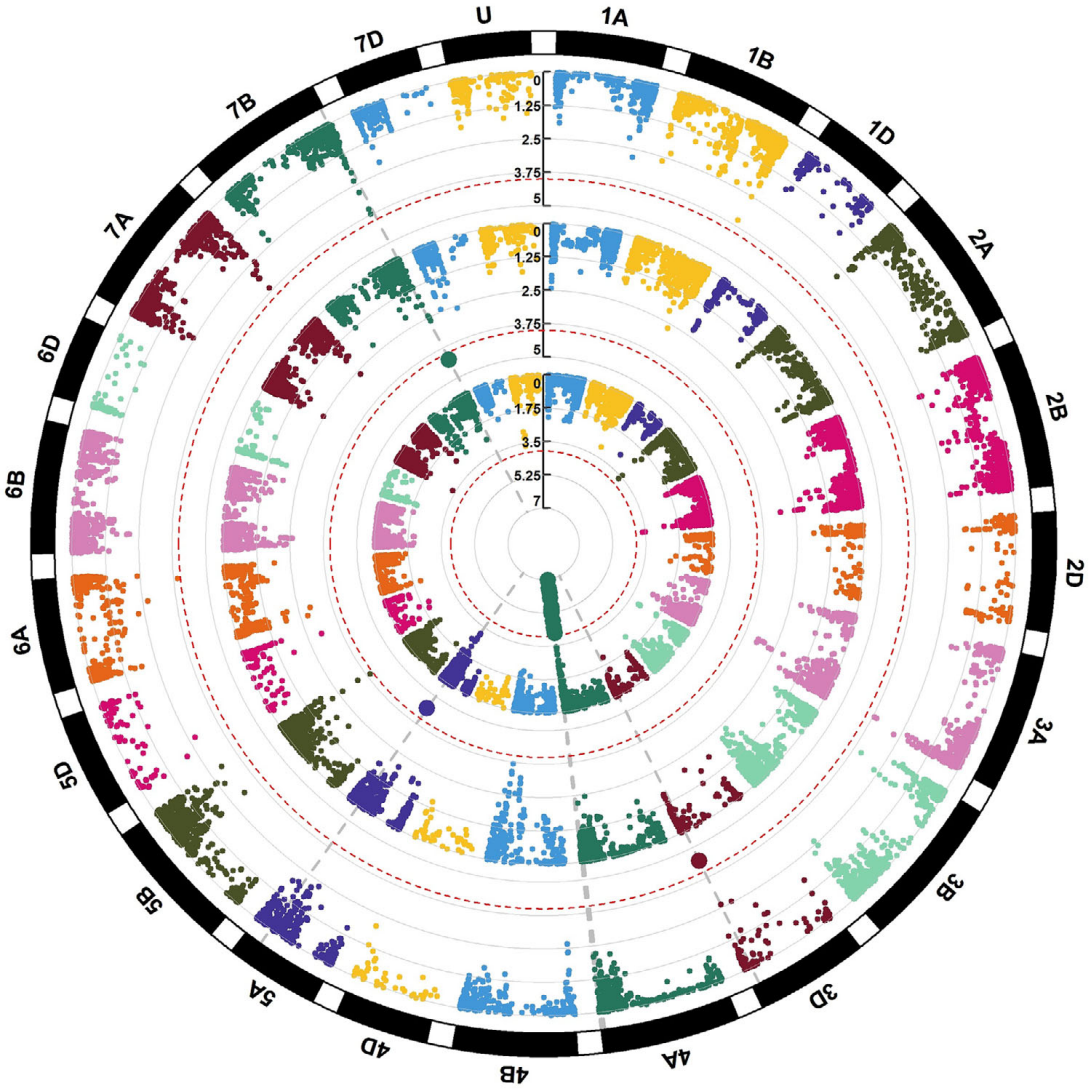
Association signals detected for *Puccinia graminis* f. sp. *tritici* races with mean difference between TKTTF isolates from Ethiopia and Germany (track 1), mean difference between TTKSK and TTKTT (track 2), and mean difference between TTKSK under cold and normal temperatures (track 3). Track numbering is from inside outward. The threshold p‐value (p = .0001) was indicated by a dotted red line for each track

For the mean infection type difference between TKTTF isolates from Ethiopia and Germany, strong association signals were detected on chromosome 4AL both with SLMM and MLMM models (Table 3 and 4; Figure 4 track 1). This result is expected since the two isolates differ for virulence to Sr7a, which is located on chromosome arm 4AL. For the difference between TTKSK and TTKTT races, association signals were detected on chromosomes 5AL and 7BL (Table 3; Figure 4 track 2), but only the latter MTA was picked by MLMM (Table 4). A single MTA on chromosome on chromosome 3DL was obtained for the mean difference of infection responses of race TTKSK under normal and cold temperature (Table 3; Figure 4 track 3) but an MTA on chromosome 3AL was found significant with the MLMM (Table 4).

**TABLE 3:** Marker‐trait associations (MTAs) detected for the mean difference in infection type between TKTTF‐ETH and TKTTF‐GER, TTKSK (NT) and TTKTT, and TTKSK (NT) and TTKSK (CT) using SLMM

Response‐difference^a^	SNP	Chrom	Map position (Mbp)	P‐value	MAF	FDR_P‐value	R^2^
TKTTF‐ETH‐GER	S4A_PART2_290628489	4A	743.18	1.17×10^−7^	0.47	0.00	0.07
TKTTF‐ETH‐GER	S4A_PART2_290053290	4A	742.61	3.19×10^−7^	0.47	0.00	0.06
TKTTF‐ETH‐GER	S4A_PART2_281141168	4A	733.70	5.40×10^−7^	0.50	0.00	0.06
TKTTF‐ETH‐GER	S4A_PART2_280264506	4A	732.82	1.02×10^−6^	0.48	0.00	0.06
TKTTF‐ETH‐GER	S4A_PART2_280212255	4A	732.77	3.14×10^−6^	0.50	0.01	0.05
TKTTF‐ETH‐GER	S4A_PART2_280270591	4A	732.83	4.21×10^−6^	0.48	0.01	0.054
TKTTF‐ETH‐GER	S4A_PART2_289908604	4A	742.46	5.50×10^−6^	0.47	0.01	0.05
TKTTF‐ETH‐GER	S4A_PART2_290196354	4A	742.75	6.18×10^−6^	0.46	0.01	0.05
TKTTF‐ETH‐GER	S4A_PART2_290030764	4A	742.59	1.67×10^−5^	0.46	0.02	0.04
TKTTF‐ETH‐GER	S4A_PART2_286223035	4A	738.78	3.72×10^−5^	0.50	0.03	0.04
TKTTF‐ETH‐GER	S4A_PART2_271952636	4A	724.51	6.93×10^−5^	0.37	0.07	0.04
TTKSK‐NT‐TTKTT	S5A_PART2_153133271	5A	409.68	3.51×10^−5^	0.08	0.27	0.07
TTKSK‐NT‐TTKTT	S7B_PART2_276986365	7B	730.81	5.68×10^−5^	0.08	0.26	0.07
TTKSK‐NT‐CT	S3D_PART2_97432482	3D	573.67	3.67×10^−5^	0.05	0.33	0.07

^a^ TKTTF‐ETH‐GER, difference between TKTTF‐ETH and TKTTF‐GER; TTKSK‐NT‐TTKTT, TTKSK under normal temperature and TTKTT; TTKSK‐NT‐CT, TTKSK under normal and cold temperatures.

**TABLE 4:** Marker‐trait associations (MTAs) detected for the mean difference in infection type between TKTTF‐ETH and TKTTF‐GER, TTKSK (NT) and TTKTT, and TTKSK (NT) and TTKSK (CT) using MLMM

Response difference^a^	SNP	Chrom	Position (Mbp)	P‐value	FDR_P‐value
TKTTF‐ETH‐GER	S4A_PART2_290628489	4A	743.18	1.74×10^−8^	0.00
TKTTF‐ETH‐GER	S7A_PART1_62187944	7A	62.19	7.94×10^−5^	0.36
TTKSK ‐NT‐ TTKTT	S7B_PART2_276986365	7B	730.81	1.29446×10^−5^	0.12
TTKSK NT‐CT	S3A_PART2_249803574	3A	703.91	9.80682×10^−5^	0.64

^a^ TKTTF‐ETH‐GER, difference between TKTTF‐ETH and TKTTF‐GER; TTKSK‐NT‐TTKTT, TTKSK under normal temperature and TTKTT; TTKSK‐NT‐CT, TTKSK under normal and cold temperatures.

#### 3.2.2 Principal component analysis‐based GWAS

We examined whether common stem rust resistance chromosome regions can be identified for the stem rust pathogen races via a PCA‐based GWAS approach. The 1^st^, 2^nd^ and 3^rd^ principal components explained 35.92, 27.87 and 13.45% of the phenotypic variation, respectively (Supplemental Table S1). The remaining principal components each explained < 8% of the phenotypic variation, and the 1^st^ five principal components explained 90.73% of the phenotypic variation. Loading of PCs are given in Supplemental Table S1. The 1^st^ PC showed high loading values in the same direction for all *Pg*t races except for TRTTF indicating variation in this PC explained resistance against the majority of the races. Similarly, in the 2^nd^ PC with the exception of both TRTTF and TTRTF all *Pgt* races had high loadings, but the TKTTF‐ETH and TKKTP loadings were in the opposite direction from the other races. In this 2^nd^ PC, loadings of TKTTF isolates from Ethiopia and Germany were high but opposite in direction. This means PC_2_ captures variation in a composite phenotype that represents lines resistant to the TKTTF isolate from Ethiopia but susceptible to the TKTTF isolate from Germany or, vice versa. PC_3_ was characterized by high loadings of TRTTF whereas PC_4_ was characterized by high loadings for race TTKTT. PC_6_ was characterized predominantly by high TTRTF loadings whereas PC_7_ discriminated the TTKSK normal temperature from that of the TTKSK low temperature as loadings for both temperature conditions were high but in opposite directions (represents variation of lines resistant to TTKSK under normal temperature but susceptible to TTKSK under low temperature, or vice versa).

**FIGURE 5:**
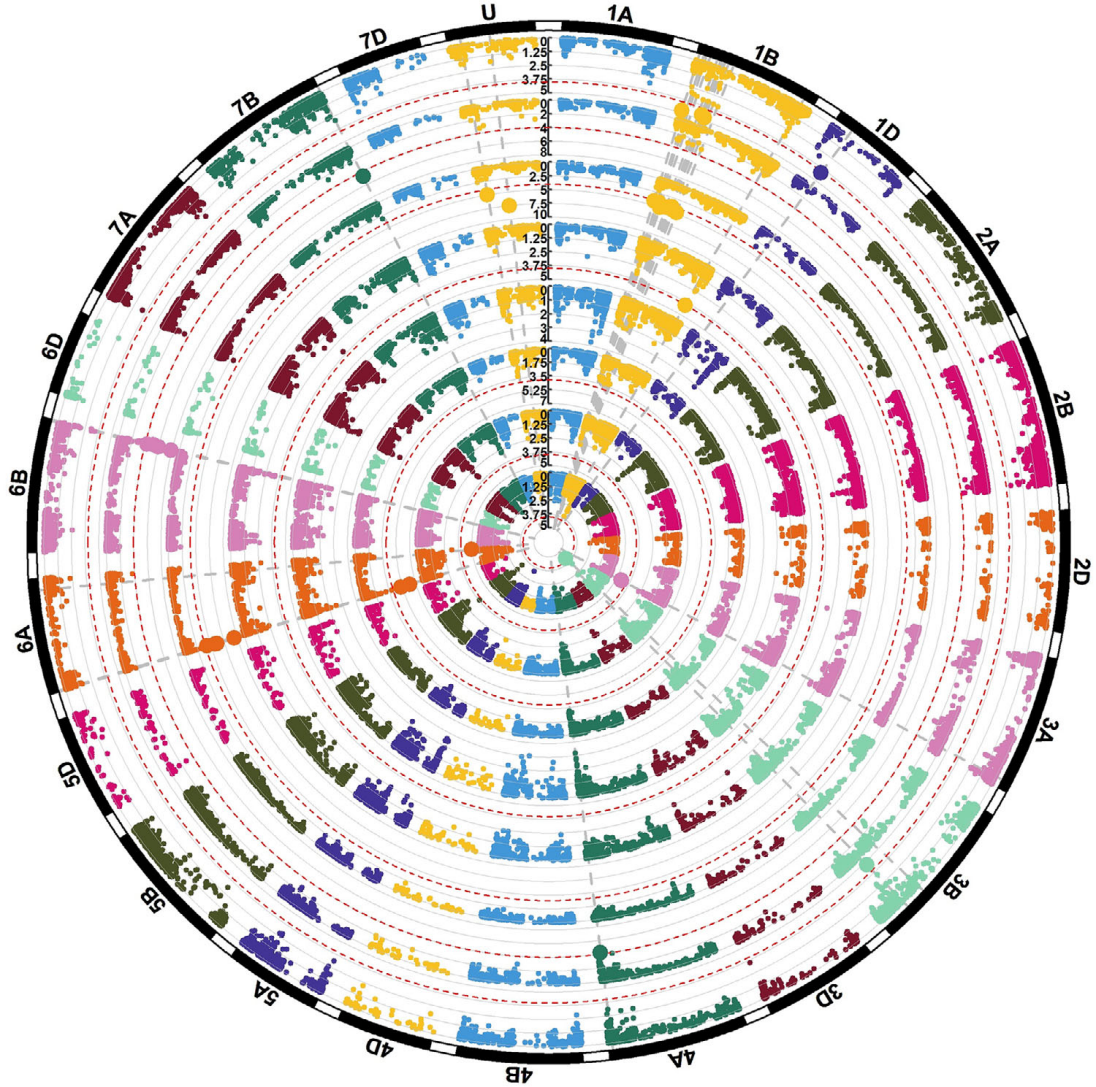
Association signals detected using PC_1_ as a trait with PC_8_ assigned to track 1 (inside track) and PC_1_ is assigned to the last track (track 8). The threshold p‐value (p = .0001) was indicated by dotted red line for each track

All significant marker‐trait associations (P < .0001) for the total of eight principal components (PCs) are shown in Table 5. Three SNPs on chromosome 1BS, in addition to one SNP on 1DS and 3BL each were significantly associated (P < .0001) with stem rust response PC_1_ (Figure 5 track 8, track counting is from inside outward). The association signals on chromosomes 1BS (1BS/S1B_PART1_156598900), 1DS for race TTRTF, and 3BL for race TTKSK (under cold temperature) were detected with single‐trait GWAS. Similarly, four SNPs on chromosome arm 6BL, and one SNP on chromosome arms 4AL and 7BL each were associated with PC_2_ (Figure 5 track 7). Association signals on chromosome arms 6BL (S6B_PART2_257935127, for race TKTTF‐ETH and TKKTP), 7BL (S7B_PART2_293794262 for race TKTTF‐GER) and 4AL (for race TTRTF) were also detected with single trait GWAS. A total of 10 and 4 SNPs on chromosomes 1BS and 6AS showed association with PC_3_ (Figure 5 track 6), respectively. The association signal on chromosome arm 6AS (S6A_PART1_3015737/S6A_PART1_3206675) was detected with single trait GWAS for stem rust resistance against race TRTTF in the region of *Sr8a*. For PC_4_, there was only one association signal with SNP S1B_PART2_187509081 (1BL) and it was not detected with single trait GWAS for any race (Figure 5 track 5). Two SNPs on chromosome arm 6AS (SNPs S6A_PART1_3015737 and S6A_PART1_6842176) showed significant associations with PC_6_ (Figure 5 track 3) and both associations were shared with PC_3_. Similarly, PC_1_ and PC_3_ shared association signals on chromosome arm 1BS with SNPs 1B_PART1_15659890, S1B_PART1_136168911 and S1B_PART1_158545386. There were three significant associations (two on chromosome arm 3AL and one on chromosome arm 6AL) for PC_7_ (Figure 5 track 2) while there was only one association signal detected for PC_8_ (Figure 5 track 1).

**TABLE 5:** Marker‐trait associations (MTAs) detected for wheat response to seven stem rust pathogen isolates using PCA‐GWAS

PCA	SNP	Chrom	Position	P‐value	FDR_P‐value	R^2^
PC1	S1B_PART1_15659890	1B	15.66	1.63×10^−5^	0.057	0.06
PC_1_	S1B_PART1_158545386	1B	158.55	1.63×10^−5^	0.057	0.06
PC_1_	S3B_PART2_212174230	3B	660.33	1.88×10^−5^	0.057	0.05
PC_1_	S1B_PART1_136168911	1B	136.17	2.63×10^−5^	0.06	0.05
PC_1_	S1D_PART1_105157874	1D	105.16	7.10×10^−5^	0.128	0.05
PC_2_	S6B_PART2_257935127	6B	710.01	1.37×10^−8^	0	0.12
PC_2_	S6B_PART2_257987924	6B	710.07	1.11×10^−6^	0.005	0.08
PC_2_	S6B_PART2_265342731	6B	717.42	7.07×10^−6^	0.021	0.07
PC_2_	S7B_PART2_293794262	7B	747.62	2.02×10^−5^	0.046	0.06
PC_2_	S6B_PART2_256708803	6B	708.79	5.15×10^−5^	0.087	0.05
PC_2_	S4A_PART2_269860502	4A	722.42	5.80×10^−5^	0.087	0.05
PC_3_	S6A_PART1_3206675	6A	3.21	3.55×10^−10^	0	0.12
PC_3_	SUN_231626593	UN	231.63	3.24×10^−8^	0	0.09
PC_3_	S6A_PART1_3015737	6A	3.02	6.95×10^−7^	0.002	0.07
PC_3_	S6A_PART1_3691352	6AS	3.69	1.50×10^−6^	0.003	0.07
PC_3_	SUN_71041528	UN	71.04	9.32×10^−6^	0.016	0.06
PC_3_	S1B_PART1_74249006	1B	74.25	1.04×10^−5^	0.016	0.06
PC_3_	S1B_PART1_209129922	1B	209.13	1.43×10^−5^	0.018	0.05
PC_3_	S1B_PART1_180406818	1B	180.41	2.10×10^−5^	0.024	0.05
PC_3_	S1B_PART1_136168911	1B	136.17	2.64×10^−5^	0.024	0.05
PC_3_	S1B_PART1_115449622	1B	115.45	2.98×10^−5^	0.024	0.05
PC_3_	S1B_PART1_146796395	1B	146.80	2.98×10^−5^	0.024	0.05
PC_3_	S6A_PART1_6842176	6A	6.84	5.76×10^−5^	0.041	0.05
PC_3_	S1B_PART1_39577562	1B	39.58	6.78×10^−5^	0.041	0.05
PC_3_	S1B_PART1_53250247	1B	53.25	7.00×10^−5^	0.041	0.05
PC_3_	S1B_PART1_15659890	1B	15.66	7.24×10^−5^	0.041	0.05
PC_3_	S1B_PART1_158545386	1B	158.55	7.24×10^−5^	0.041	0.05
PC_4_	S1B_PART2_187509081	1B	626.23	8.80×10^−5^	0.795	0.06
PC_6_	S6A_PART1_3015737	6A	3.02	5.97×10^−7^	0.005	0.09
PC_6_	S6A_PART1_6842176	6A	6.84	8.17×10^−6^	0.037	0.07
PC_7_	S6A_PART2_102668084	6A	555.11	1.10×10^−5^	0.096	0.08
PC_7_	S3A_PART2_249803574	3A	703.91	2.94×10^−5^	0.096	0.07
PC_7_	S3A_PART2_249742725	3A	703.85	3.19×10^−5^	0.096	0.07
PC_7_	S5B_PART2_20009089	5B	471.38	0.000157	0.356	0.06
PC_8_	S3B_PART2_94939208	3B	543.09	6.65×10^−5^	0.47	0.07

## 4 DISCUSSION

We report that 80% of North American spring wheat advanced breeding lines and cultivars are susceptible as seedlings to emerging *Pgt* race TTRTF. Though this level of susceptibility is lower than that for the Ug99 race group, the broad susceptibility to TTRTF suggests that this race could cause devastating wheat yield losses if it were to arrive in North America. However, we detected modest levels of susceptibility (25.81%) to the new race TKKTP from Germany suggesting that North American spring wheat is not as vulnerable to this race. The abundant resistance to race TKKTP in North American spring wheat is likely conferred by *Sr11* and *Sr7a*.

Race TTRTF is a relatively newly emerged stem rust pathogen race. A key objective of this study was to identify QTL effective to this emerging race. Association signals were consistently detected on chromosome 1BS (15.66 Mbp), 2BS (23.97 Mbp), 2A (30.33 Mbp) and 4AS (268.33 Mbp). Out of these associations, the strongest signal was on chromosome 1BS (P‐value of 1.12×10^−11^). The resistance gene *Sr31* that has been widely used in several breeding programs is located on a 1RS translocation that replaces 1BS. Given that *Sr31* is effective against TTRTF as confirmed by the wheat differential set (Olivera et al., 2019; Wanore, Chala, Shikur, Hodson, & Szabo, 2019), and that lines with *Sr31* are present in the panel (Bajgain et al., 2015a), the resistance against race TTRTF on chromosome 1BS is most likely due to the effect of *Sr31*. Significant association signals were detected on the short arm of chromosome 1DS both for races TTRTF and TRTTF at different locations. Major known stem rust resistance genes on chromosome arm 1DS are *Sr33*, *Sr45*, *Sr50* and *SrTA166*2 (Yu et al., 2014). From cluster analysis of stem rust pathogen isolates using a custom SNP chip by Olivera et al. (2019), race TTRTF is genetically related to race TRTTF as both are in Clade III with TTRTF in sub‐clade III‐B, and with TRTTF in sub‐clade III‐A. Although the two isolates had been collected from different regions (TTRTF from Georgia and TRTTF from Yemen), a recent study by Li et al. (2019) indicated that *Pgt* lineages originated from distinct geographic areas can share a large portion of their genomes. Previously, association signals were reported for resistance against race TRTTF on chromosome 1B (43.9–64.9 cM) by Bajgain et al. (2015a). However, we found no QTL in common between responses to TRTTF and TTRTF that suggests that, even for related isolates, resistance can be quite specific. At the time of writing this manuscript, our result was the first report of chromosome regions associated with resistance to race TTRTF based on association mapping. However, of the QTL found effective to race TTRTF in the current study, QTL on chromosomes 2BS, 2A, and 4AS do not correspond to previously characterized QTL in this germplasm. Thus, these QTL need to be validated before utilization in wheat breeding.

Another objective of this study was to identify chromosome regions associated with resistance against other *Pgt* races. Resistance against the two closely related races TKTTF and TKKTP was identified on chromosome arm 6BL (∼93.32 cM). The major known gene that confers stem rust resistance on chromosome arm 6BL is *Sr11* (Nirmala et al., 2016; Sears, 1966), and is effective to both races. These two races, TKTTF and TKKTP, are genetically different from members of the Ug99 race group. Race TKTTF is avirulent to stem rust resistance genes *Sr11*, *Sr24*, and *Sr31* (Olivera et al., 2015). However, it is virulent on gene *SrTmp* that is carried by the widely adopted bread wheat variety Digalu whose susceptibility to TKTTF resulted in stem rust epidemics in Ethiopia in 2013–2014. Although the race TKTTF isolate that was obtained from Germany was phenotypically different from the Ethiopian TKTTF isolate (the former is virulent to *Sr7a*, *Sr45* and *SrTt‐3* genes; Olivera et al., 2017), the association signals detected on chromosome arms 6BL and 7BL were common for both German and Ethiopian isolates of race TKTTF. Previously a significant QTL was reported on chromosome arm 6BL for resistance against the Ethiopian TKTTF isolate in both with GWAS and biparental analysis (Bajgain et al., 2015a; Nirmala et al., 2016). Therefore, markers linked to *Sr11* can be used to track resistance against both German and Ethiopian TKTTF isolates in addition to race TKKTP. However, there were association signals specific to particular isolates. For instance, the association signal on chromosome arm 4AL was consistently detected for response against the Ethiopian isolate of race TKTTF whereas an association signal was detected on chromosome arm 2BL for response to the German isolate. There was also an association signal for race TKKTP (586.91 Mbp) on chromosome 2BL at a different position from that of TKTTF (692.62 Mbp) from Germany. There are at least seven QTL linked to stem rust resistance on chromosome arm 2BL in addition to major stem rust resistance genes such as *Sr9h*, *Sr28* and *Sr47* (Rouse et al., 2014a; Yu et al., 2014). Although no known major gene has been reported on chromosome arm 7BL, stem rust resistance QTLs have previously been reported (Bansal et al., 2008; Yu et al., 2014).

There are chromosome regions associated with resistance against races TKKTP, TKTTF (from Ethiopia) and TTRTF on chromosome 4AL. Although TKKTP and TKTTF (from Ethiopia) QTL shared the same region, the SNP associated with TTRTF resistance is located a minimum of 465 Mbp away from this position. There are at least four stem rust resistance QTL on chromosome 4AL, and *Sr7a* has been reported on 4AL (Turner, Jin, Rouse, & Anderson, 2016). *Sr7a* is effective to TKTTF from Ethiopia but not TKTTF from Germany.

Previous mapping work with SNPs and SSR markers located TRTTF resistance on chromosome arm 6AS postulated as *Sr8a* (Bajgain et al., 2015a; Guerrero‐Chavez, Glover, Rouse, & Gonzalez‐Hernandez, 2015; Hiebert, Rouse, Nirmala, & Fetch, 2016; Nirmala et al., 2017). Similarly, in the current study, a strong association signal was detected on chromosome 6AS suggesting the involvement of *Sr8a* as it is the only major stem rust resistance gene present in bread wheat effective to TRTTF on chromosome arm 6AS. There may be other genes that confer TRTTF resistance, but their allele frequency in this mapping panel could be lower than the necessary frequency (>5%) to enable detection. It should be noted that the exclusion of low frequency alleles from GWAS analysis is mainly due to lack of resolution to detect association signals for functional alleles present in low frequency (usually they are ignored in analysis). Otherwise, rare alleles could significantly explain genetic variations in phenotypic traits (Alqudah, Sallam, Baenziger, & Börner, 2020). These rare variants that are unique to a specific group of individuals and would only be detected in GWAS if the frequency was greater (Brachi et al., 2011).

The unique technical components of this study were the association mapping of differences between phenotypic mean responses to pairs of *Pgt* races (e.g., the mapping of difference between race TTKSK responses at two temperature regimes) and the PCA‐based association analysis. The identified difference in between race TKTTF isolates from Germany and Ethiopia on 4AL was expected because of the previously characterized difference in avirulence to *Sr7a* between these two isolates. However, we also detected a QTL on chromosome arm 7AS based on phenotypic mean difference. The 7AS QTL was not detected using the data from either isolate of TKTTF, individually. Similarly, for the difference between races TTKSK and TTKTT, we identified a QTL on chromosome arm 7BL that was not detected when mapping the responses of these two races individually. This implies that mapping using phenotypic mean difference can be a useful way to identify cryptic virulence changes between closely related isolates. Finally, when mapping the mean difference in responses to race TTKSK at normal and low temperature, we identified a QTL on chromosome arm 3AL that was not detected with either temperature regimes. Only a single association signal (3B, genetic map position 58.7–60.30 cM) co‐localized in both the normal and cold temperatures. Previous studies showed that there are both major stem rust resistant genes and QTL on chromosome 3BS. For instance, one of the previously known genes, *Sr12* is a seedling stem rust resistance gene that resides in the centromeric region of chromosome 3B (flanked by SNP markers *IWA6086* at consensus genetic position 82.85 cM and *IWA4613* at consensus genetic position 85.83 cM; Hiebert et al., 2016). Bajgain et al. (2015a) also reported SNPs that were significantly associated with resistance against TTKSK on chromosome 3B (67.5–76.9 cM) near the *Sr12* locus. The 3AL QTL detected in mapping the difference between low and normal temperature was also detected with PCA‐GWAS for PC_7_ which is characterized by individuals resistant for TTKSK under normal temperature condition but susceptible under low temperature, or vice versa. This QTL was not identified in the linearized infection type data for TTKSK at low temperature, likely because the response was masked by the QTL on 3B (*Sr12*) and other QTL detected at this temperature. Therefore, PCA‐GWAS proved useful for identifying QTL that would not have been detected using mixed model mapping and phenotyping at normal temperatures. It is worthwhile to mention that the known major genes (*Sr7a, Sr8a, Sr11, Sr12*) detected in this study are common in the current mapping panel with the exception of *Sr31*, which is present in the panel at a lower frequency (Edae et al., 2018). Going forward, association mapping of phenotypic differences may be valuable for elucidating the genetics of genotype by environment interactions including those mediated by temperature. We also validated the use of PCA‐GWAS approach to confirm MTAs identified with other approaches especially when highly correlated traits are used for GWAS (Ried et al., 2016). Interestingly, all association signals in the region of major genes (*Sr7a, Sr8a, Sr11, Sr12*, and *Sr31*) and other strong association signals that were detected with standard GWAS were also detected with the PCA‐GWAS with the 1^st^ two PCs. This implies that there is high concordance between PCA‐GWAS and standard GWAS methods, and the PCA‐GWAS can be used especially to identify strong association signals.

In conclusion, for all *Pgt* isolates evaluated in this study two or more association signals were detected implying resistance against these *Pgt* races is multigenic. Although there are some QTL regions shared across the races, none of the association signals were detected in response to all races. We also found that there was a case where a major gene that was ineffective to an older virulent race but effective to newly emerged virulent race (e.g., *Sr31* is effective to the new race TTRTF but not to TTKSK). This suggests pyramiding major genes is still a useful strategy to mitigate dangerous stem rust pathogen races including recently‐emerged races.

## AUTHOR CONTRIBUTIONS

EE and MR conceived the experiment. MR generated greenhouse phenotypic data and obtained the funding. EE conducted SNP calling and drafted the manuscript.

## Supporting information

Supplemental Figure S1. Distribution of genotype‐by‐sequencing markers on wheat chromosomes.

Supplemental Figure S2. Clusters of the spring wheat lines in the association panel with principal component analysis.

Supplemental Figure S3. Linkage disequilibrium decay curve for chromosome arm 6BL.

Supplemental Figure S4. Linkage disequilibrium decay curve for chromosome arm 7BL.

Supplemental Figure S5. Linkage disequilibrium heatmap for chromosome arm 6BL.

Supplemental Figure S6. Linkage disequilibrium heatmap for chromosome arm 7BL.

Supplemental Table S1. Loadings of Prncipal components for all *Pgt* races evaluated in the study and proportion of variace explained by each principal component

Supplemental Table S2. Phenotypic and genotypic raw data for SNPs that showed significant associations in this study

## Data Availability

The GBS raw sequence data can be accessed with BioProject ID PRJNA641969 from NCBI (National Center for Biotechnology Information) website at http://www.ncbi.nlm.nih.gov/bioproject/641969. However, both raw phenotypic and genotypic data of the SNPs that showed significant associations for all races evaluated in this study were given in Supplemental Table S2.
